# EGFR-Targeted Therapeutics: Focus on SCCHN and NSCLC

**DOI:** 10.1100/tsw.2008.117

**Published:** 2008-09-21

**Authors:** Martin Sattler, Oyewale Abidoye, Ravi Salgia

**Affiliations:** ^1^ Department of Medical Oncology, Dana-Farber Cancer Institute, Harvard Medical School, Boston, USA; ^2^ Brigham and Women's Hospital, Harvard Medical School, Boston, USA; ^3^ Department of Medicine, University of Chicago, USA

**Keywords:** cetuximab, epidermal growth factor receptor, Erbitux, head and neck cancer, lung cancer, targeted therapy

## Abstract

Cancers of the head and neck and of the lung are associated with high morbidity and mortality rates that have remained relatively unchanged for more than 3 decades, despite advances in radiation therapies and chemotherapies over the same time. It is generally believed that the efficacy of standard therapy regimens has reached a plateau for these cancers. The discovery of specific aberrant molecular signaling pathways in solid tumors has afforded promising new directions for newer “targeted” cancer therapeutics. Among these, the epidermal growth factor receptor (EGFR) shows promise as a therapeutic target. Clinical studies have demonstrated that this targeted approach provides clinically meaningful benefit. This article reviews EGFR-targeted therapies in use and in development, with a focus on the role of EGFR in the pathophysiology of head and neck and lung cancer, and new concepts being investigated to improve outcomes with these agents.

## INTRODUCTION

Approximately 90% of cancers of the head and neck are squamous cell carcinomas arising from the epithelial lining of the nose, mouth, and throat. Approximately two-thirds of patients present with stage III or IV squamous cell carcinoma of the head and neck (SCCHN), of whom <30% are cured with standard treatments. The latter includes surgery with adjuvant cisplatin-based chemoradiotherapy for resectable, locally advanced SCCHN, and chemotherapy and/or radiation for unresectable disease[1].

Non–small cell lung cancer (NSCLC) comprises 75–85% of newly diagnosed lung cancers. Although the prognosis is much better with early detection, over 70% of NSCLC patients present with advanced disease and a 5-year survival rate overall for NSCLC is 16%. For early-stage or locally advanced lung cancer, surgery is the most effective treatment, and combined chemotherapy is the standard adjuvant approach[2]. For stage III/IV NSCLC, platinum-based combined chemotherapy is the current standard of care[3].

Despite substantial laboratory and clinical efforts, patient survival from state-of-the-art treatments appears to have plateaued for both of these tumor types. Targeted agents, such as those specific for the epidermal growth factor receptor (EGFR), represent a new paradigm in cancer therapy and have shown therapeutic promise in a wide range of tumor types, including SCCHN and NSCLC.

## EGFR SIGNALING IN NSCLC AND SCCHN: AN IDEAL THERAPEUTIC TARGET

EGFR is a member of the ErbB, or epidermal growth factor (EGF), family of transmembrane receptor tyrosine kinases (RTKs), which includes four related receptor proteins (EGFR/ErbB1/HER1, ErbB2/Neu/HER2, ErbB3/HER3, and ErbB4/HER4). EGFR is expressed on many normal cell types, including epithelial and mesenchymal cells, and, through a complex network of ligands and intracellular signaling pathways, is involved in the regulation of cell proliferation, survival, adhesion, migration, and differentiation[4]. EGFR also mediates processes involved in tumor growth and progression, including angiogenesis, tumor cell proliferation, metastasis, and antiapoptosis[4].

EGFR is highly expressed in 80–100% of head and neck tumors and in 40–80% of NSCLC[5,6]. Increased expression of EGFR is often associated with poor prognosis, especially in head and neck cancer; however, this association is observed less frequently in NSCLC[6].

Numerous agents that target EGFR have been extensively studied, with some highly encouraging clinical results. Currently, the most clinically advanced strategies target either the EGFR extracellular/ligand-binding domain with monoclonal antibodies (MAbs) or the EGFR intracellular/tyrosine kinase domain with small-molecule tyrosine kinase inhibitors (TKIs).

EGFR ligand binding triggers receptor dimerization, affording autophosphorylation of tyrosine residues in the cytoplasmic C-terminal tail and thereby activating a network of intracellular signaling pathways. The complex EGFR signal transduction pathway involves the Ras/MAPK cascade, phosphatidyl inositol 3-kinase (PI3K), signal transducer and activator of transcription (STAT), and downstream protein kinase C (PKC)[4] (Fig. 1 — click the link to view the animated figure. http://www.thescientificworld.com/supplements/2008.117/EGFR.swf)

## ANTI-EGFR MONOCLONAL ANTIBODIES

Cetuximab (Erbitux®), a chimeric, human-murine IgG1 MAb, was the first anti-EGFR MAb to receive FDA approval. It is now indicated for use in both chemotherapy-refractory metastatic colorectal cancer (mCRC) and advanced, recurrent, or metastatic SCCHN (Erbitux® [prescribing information], ImClone, Princeton, NJ; 2007). In 2006, another anti-EGFR antibody, panitumumab (Vectibix®), was approved for mCRC on progression while on or following standard chemotherapy (Vectibix® [prescribing information], Amgen, Thousand Oaks, CA; 2007). Other investigational anti-EGFR mAbs include matuzumab (EMD72000), zalutumumab (HuMax-EGFr), MDX-447, hR3 (nimotuzumab), and Mab806.

### Cetuximab

Cetuximab binds to the extracellular EGFR domain, thus blocking ligand binding. Cetuximab may also inhibit EGFR activity through several mechanisms: (1) by inducing receptor internalization and reducing the number of exposed EGFR molecules, (2) through antibody-dependent cellular cytotoxicity (ADCC) or complement-dependent cytotoxicity (CDC)[7], and (3) by enhancing the cytotoxic effects of chemotherapeutic agents and radiotherapy[8].

Cetuximab is associated with adverse events, acneiform rash being the most common (Erbitux® [prescribing information], ImClone, Princeton, NJ; 2007). The intensity of the rash, which resolves without scarring on therapy termination, can be predictive of clinical response. A randomized study of 166 patients with mCRC who exhibited no or mild skin rashes in response to the standard cetuximab dose showed that the dose could be increased, with the resulting skin rash intensity associated with improvements in tumor response rate[9]. Other commonly reported adverse events (e.g., diarrhea, asthenia, nausea) are manageable and generally do not overlap with those associated with chemotherapy or radiation.

## Cetuximab in NSCLC

Preliminary clinical studies in patients with advanced NSCLC have been reported with cetuximab monotherapy or in combination with chemotherapeutic agents, both in chemotherapy-exposed and chemotherapy-naive patients.

A phase 2 study investigated cetuximab monotherapy in NSCLC patients with recurrent or progressive/metastatic disease following at least one line of chemotherapy[10]. While patients with EGFR expression achieved a response rate of 5% (three of 60 patients), the overall response rate (ORR) was 4.5% and stable disease (SD) was obtained in 30.3% of patients (n = 66).

Randomized phase 2 studies have reported increased activity and acceptable safety profiles of various cetuximab-chemotherapy combinations when compared with chemotherapy alone for the treatment of recurrent or advanced NSCLC[11,12,13]. Based on these encouraging early clinical data, a number of clinical trials have been designed to examine different cetuximab-chemotherapy regimens, including a phase 3 study (FLEX) that has first-line cisplatin/vinorelbine ± cetuximab in more than 1000 advanced NSCLC patients (www.clinicaltrials.gov). The FLEX study was recently presented at the 2008 American Society of Clinical Oncology (ASCO) meeting, and showed that cetuximab added to first-line chemotherapy with cisplatin/vinorelbine (median overall survival 11.3 months, 1-year survival 47%) demonstrated superior overall survival compared to chemotherapy alone (median overall survival 10.1 months, 1-year survival 42%) in patients with advanced EGFR-expressing NSCLC[14]. Also, another phase 3 clinical trial was presented at the International Association for the Study of Lung Cancer (IASLC) meeting in 2007 for carboplatin/taxane with or without cetuximab[15], and this did not meet the primary end point of overall survival difference.

Cetuximab also appears to be very effective when combined with radiation therapy, both for SCCHN (described below) and potentially for NSCLC. Synergistic enhancement of radiation has been documented in NSCLC cell lines that were pretreated with either cetuximab or erlotinib[16]. In the clinical setting, the feasibility of combining cetuximab with chemoradiation (CRT) was demonstrated in a study of 93 patients with stage III NSCLC who achieved a 62% response rate and a 12-month overall survival (OS) of 68%[17].

## Cetuximab in SCC of the Head and Neck

Treatment of SCCHN with cetuximab is efficacious in combination with chemotherapy or radiation[1,18]. Most notably, cetuximab was recently approved by the FDA for treatment of SCCHN in combination with radiotherapy for locally or regionally advanced SCCHN, and as monotherapy for recurrent or metastatic SCCHN refractory to platinum-based therapy (Erbitux® [prescribing information], ImClone, Princeton, NJ; 2007). Approval of cetuximab in the treatment of SCCHN was based on results of a large international phase 3 study involving 424 patients with locoregionally advanced SCC of the oropharynx, hypopharynx, or larynx who were randomized to receive either high-dose radiation alone for 6–7 weeks or radiation plus weekly cetuximab and followed for 5 years[19]. Median OS was significantly longer with the addition of cetuximab — 49.0 months in the cetuximab plus radiation group vs. 29.3 months in the radiation-only group ($p$ = 0.03) — although it must be noted that chemoradiation, and not radiation therapy alone, is the standard treatment for locally advanced SCCHN. Addition of cetuximab to radiotherapy did not adversely affect the patients' quality of life (QOL)[20]. Currently, cetuximab remains the only molecular targeted therapy clearly demonstrated to improve patient survival in SCCHN significantly when combined with radiation or platinum-based chemotherapy.

Following phase 1/2 studies that showed the feasibility of adding cetuximab to platinum-based chemotherapy, this combination was evaluated as first-line treatment in a phase 3 trial involving 442 evaluable patients with metastatic/recurrent SCCHN. The median OS was significantly prolonged to 10.1 months in the chemotherapy plus cetuximab arm vs. 7.4 months in the chemotherapy plus placebo arm ($p = 0.036$), and interim safety analysis showed no changes in the platinum-based chemotherapy toxicity profile[21]. This cetuximab-chemotherapy combination is the first systemic treatment to show a survival benefit over platinum-based chemotherapy in this setting.

A cetuximab/cisplatin/radiation therapy triple-combination regimen will be tested in a phase 2 study (RTOG 0522) to assess its efficacy in patients with stage III or IV SCCHN (www.clinicaltrials.gov). Although this combination yielded encouraging data (75% OS and 56% progression-free survival [PFS]) in an earlier study of 22 patients[22], the study was discontinued due to two deaths and one case each of atrial fibrillation, myocardial infarction, and bacteremia. Since then, another study investigating cetuximab combined with alternating chemotherapy and radiation therapy has shown objective responses in all of the first 16 patients with available data (total N = 24)[23]. However, this trial also showed a new, previously unreported, desquamating type of skin toxicity associated with radiation therapy.

An open-label multicenter study of 103 patients with recurrent/metastatic SCCHN refractory to platinum-based chemotherapy demonstrated the feasibility of cetuximab for SCCHN. Cetuximab was tested as a single agent until disease progression, on which 53 of the patients received cetuximab plus platinum combination therapy. Results during the single-agent phase compared to those during the combination-therapy phase included ORR of 13 vs. 0%, disease control rate (DCR) of 46 vs. 26%, and median time to progression (TTP) of 70 vs. 50 days[24]. The response was comparable to that seen with cetuximab plus platinum combination regimens in similar settings.

## Other Anti-EGFR Antibodies in SCCHN and NSCLC

Other anti-EGFR MAbs lag behind cetuximab in clinical development, with zalutumumab and panitumumab currently the most advanced in the development pipeline. Zalutumumab (HuMax-EGFr™; Genmab) is being evaluated in a phase 3 study as monotherapy for incurable SCCHN, as well as a phase 1 and 2 study in combination with chemoradiation for advanced SCCHN. Panitumumab is being tested in phase 2 (PARTNER) and phase 3 (SPECTRUM) studies in combination with chemotherapy, and in a phase 2 study (PRISM) as second-line monotherapy for recurrent/metastatic SCCHN (www.clinicaltrials.gov).

As more molecular signatures are performed for SCCHN and NSCLC in the context of EGFR, it will also be important to determine the relationship of EGFR response and K-ras mutations. As has been determined recently for colon cancer, antibody therapy against EGFR is more effective in wild-type K-ras–containing tumors.

## EGFR TKIs

TKIs act by binding to the intracellular EGFR tyrosine kinase domain and by inhibiting receptor phosphorylation and downstream signaling. The two best-studied TKIs are the low-molecular-weight oral quinazoline compounds gefitinib (Iressa®/ZD1839) and erlotinib (Tarceva®/OSI-774). Other TKIs include two dual kinase inhibitors, lapatinib (Tykerb®/GW572016) and AE788, and several second-generation TKIs under investigation (e.g., PKI166, EKB569, canertinib [CI-1033], HKI-272, HKI-357, CL-387.785, and BIBW 2992[25]). In clinical trials, gefitinib and erlotinib are generally well tolerated. As with cetuximab, an acne-like skin rash is a common adverse event for both agents and the rash appears to correlate with response[26,27]. Diarrhea, occurring with a similar frequency as skin rash, is also a common adverse event and may be dose limiting[26,27]. Reversible elevations of serum transaminases have been reported with PKI 166 and GW572016[28].

## TKIs in NSCLC

Gefitinib (Iressa®) originally received accelerated approval as monotherapy for advanced refractory NSCLC based on two phase 2 trials (IDEAL 1 and 2); however, it failed to show an additional OS advantage in combination with platinum-based first-line regimens for NSCLC in phase 3 trials (INTACT 1 and INTACT 2)[29]. In addition, the drug failed to show a significant survival benefit over best-supportive care in a placebo-controlled, randomized phase 3 study (the Iressa Survival Evaluation in Lung Cancer study [ISEL]), although the highest differences in objective response rate for gefitinib vs. placebo were seen among never-smokers, women, patients with adenocarcinoma, and patients of Asian origin[30]. As a result, in the U.S., gefitinib is now indicated only for patients who are currently benefiting or have previously benefited from gefitinib treatment (Iressa® [prescribing information], AstraZeneca, Wilmington, DE; 2005). Also, a phase 3 trial of maintenance gefitinib (or placebo) after concurrent chemoradiotherapy and docetaxel consolidation in stage III NSCLC was reported, with median survival of 23 months for gefitinib (n = 118) and 35 months for placebo (n = 125; two-sided $p = 0.013$). The toxic death rate was 2% with gefitinib compared with 0% for placebo[31].

Erlotinib (Tarceva®) is currently approved for treatment of locally advanced or metastatic NSCLC after failure of at least one prior chemotherapy regimen (Tarceva® [prescribing information], Genentech, South San Francisco, CA; 2005). It is also indicated, in combination with gemcitabine, for the first-line treatment of patients with locally advanced unresectable or metastatic pancreatic cancer.

In contrast to gefitinib, erlotinib yielded a significant survival benefit in NSCLC patients. In a randomized, placebo-controlled, double-blind, international phase 3 trial involving 731 patients with stage IIIB/IV NSCLC, OS rates were 6.7 and 4.7 months for patients receiving erlotinib (150 mg/day) or placebo, respectively ($p = 0.001$). The overall response to erlotinib was 8.9% (95% CI: 6.6–12.0%, $p < 0.001$), with a median duration of response of 34.2 weeks. The time to deterioration in patient-reported symptoms (the primary QOL analysis) also showed statistically and clinically meaningful benefit for patients randomized to erlotinib[32].

A number of trials have combined chemotherapy and/or radiation therapy with small-molecule EGFR TKIs; however, there have been no clear-cut benefits (for review, see [33]).

## TKIs in SCCHN

Experience with TKIs in head and neck cancer is limited. Results of a phase 2 study of erlotinib in combination with docetaxel and cisplatin in 47 patients with recurrent/metastatic SCCHN showed an acceptable safety profile and encouraging activity, with an ORR of 67%, DCR of 95%, median OS of 11 months, and PFS of 6.01 months[26].

Docetaxel, with or without gefitinib, is currently in phase 3 trials for the treatment of metastatic or recurrent SCCHN. Initial phase 1 and 2 studies of gefitinib or erlotinib combined with various other chemotherapies, radiotherapy, and VEGF (vascular endothelial growth factor)-targeted therapies for the treatment of SCCHN are also in progress (www.clinicaltrials.gov)

## *EGFR* MUTATIONS: A PATHWAY TO RESISTANCE AND SUSCEPTIBILITY

Different *EGFR* mutations have been associated with either resistance or susceptibility to targeted agents, especially to gefitinib and erlotinib. Resistance may be described as primary resistance, with no response to treatment prior to progression, or secondary resistance, in which an initial response is followed by disease progression, usually within 6–8 months. The latter is likely to be related to an acquired mutation, which may affect ligand binding, or to differences in drug bioavailability[34].

## Molecular Mechanisms

The discovery of somatic mutations in the tyrosine kinase domain of the *EGFR* gene in lung adenocarcinomas responding to gefitinib or erlotinib has led to a new understanding of EGFR-mediated resistance and susceptibility. In the Canadian Clinical Trials Group (NCIC CTG) BR.21 study, *EGFR* mutations were not only found in 31% of nonsmokers and 21% of current or exsmokers, but also occurred more frequently in women, patients of Asian descent, and those with adenocarcinoma[35]. Currently identified mutations that confer TKI susceptibility include exon 19 deletions, L858R, L861Q, G719A/C/S, E884K; exon 20 insertions and T790M confer resistance[36,37].

There is sufficient evidence that *EGFR* mutations can dramatically influence the response to TKI treatment. For example, in a study of first-line erlotinib in NSCLC, 38 evaluable patients with *EGFR* mutations achieved an ORR of 82%, including a complete response (CR) rate of 13%[38]. The highest response rate was observed among the 20 patients with exon 19 mutations (95%). Of the 18 patients with exon 21 mutations, 67% responded.

Eighty percent of tumors from the NSCLC patients responding to gefitinib or erlotinib harbor in-frame deletions or missense mutations in the EGFR tyrosine kinase domain compared to none in 36 refractory tumors[39]. Approximately 85–90% of these mutations occur at exons 19 and 21 near the adenosine triphosphate (ATP) cleft of the tyrosine kinase domain, where gefitinib and erlotinib compete with ATP for binding[40]. The most common mutations in *EGFR* are an exon 19 deletion that eliminates a leucine/arginine/glutamate/alanine (LREA) motif in the tyrosine kinase domain and a thymine-to-guanine transversion that results in an arginine-for-leucine substitution at position 858 (L858R) in exon 21. These *EGFR* mutations are potentially useful molecular markers that may help when selecting appropriate treatment in NSCLC[41].

The T790M mutation in exon 20 is the only EGFR resistance mutation identified to date[39]. Analyses of tumor material from three patients have shown that this mutation enables NSCLC tumors to acquired resistance to gefitinib or erlotinib.

Novel EGFR inhibitors that can inhibit EGFR L858R/T790M in gefitinib- or erlotinib-refractory cell lines are now in development. One such inhibitor is HKI-272, an irreversible oral pan-ErbB TKI with specificity for both EGFR (ErbB1) and HER2 (ErbB2)[42]. HKI-272 has shown inhibitory activity against EGFRvIII-dependent mouse lung tumors. At much lower intracellular concentrations than gefitinib and erlotinib, this agent has demonstrated potential benefit to subsets of NSCLC patients with tumors carrying the ERBB2 G776insV_G/C mutation[42].

Choong et al. reported a novel mutation at codon 884 (E884K) with a glutamic acid to lysine conversion in exon 22[37]. A patient with a known L858R NSCLC mutation exhibited disease progression while receiving erlotinib plus standard chemotherapy; however, the tumor responded dramatically to salvage therapy with gefitinib. Subsequent studies showed that the E884K mutation made the EGFR kinase more susceptible to inhibition by gefitinib, but resistant to inhibition by erlotinib. Moreover, the kinase activity of the EGFR double mutant (L858R and E884K) was more susceptible to inhibition by gefitinib than was EGFR containing either single mutation[37].

Resistance to EGFR targeting may also occur by mechanisms that involve receptor internalization or altered downstream signaling[18]. For instance, a previously gefitinib-sensitive NSCLC cell line has been shown to develop gefitinib resistance by amplification of the Met proto-oncogene, which drives ERBB3 (HER3)-dependent downstream PI3K activation[43]. There is also strong evidence that the c-Met receptor is overexpressed and sometimes mutated in most (89%) NSCLC cell lines and in 61% of tumor tissues studied, and small-molecule inhibitors of c-Met, such as SU11274, inhibit cell viability in c-Met–expressing NSCLC cells[44,45]. As a result, the c-Met receptor tyrosine kinase is emerging as a novel therapeutic target in addition to EGFR in many solid tumors, including lung cancer[46,47].

Elucidation of molecular mechanisms of EGFR resistance will allow better understanding of treatment failures with erlotinib and gefitinib, and also assist in tailoring individualized regimens for cancer patients based on their mutation status. New developments for the future will include new models to circumvent altered pharmacodynamics in patients with EGFR resistance and the development of new second-generation EGFR inhibitors.

## EGFR PATIENT SELECTION AND EGFR GENE AMPLIFICATION/POLYSOMY

EGFR gene amplification, as assessed by fluorescence *in situ* hybridization (FISH), occurs with a 30–45% frequency among patients with NSCLC[35,48]. Retrospective analyses of tumor samples from phase 2 trials showed higher response rates and longer survival times for FISH-positive cases[48,49]. A higher EGFR gene copy number was significantly associated with responsiveness to erlotinib ($p$ = 0.04) and improved OS (hazard ratio [HR] = 0.44; $p$ = 0.008). No significant improvement in survival with erlotinib was observed among patients whose tumors were FISH negative (HR = 0.85; $p$ = 0.59)[35].

Similarly, a subset analysis of patients from the ISEL trial showed longer median OS rates among patients with a higher EGFR gene copy number (8.3 vs. 4.3 months; HR = 0.78)[50]. However, the situation is complicated by discrepancies between EGFR copy number or expression at the primary tumor vs. metastatic sites, which should be considered when predicting patient responsiveness to EGFR-targeted agents[51]. Likewise, EGFR expression detected by immunohistochemical methods does not predict responsiveness of SCCHN to chemotherapy, with or without cetuximab[52]. Further studies, perhaps involving more quantitative methods and subcellular targets, will be needed to elucidate the exact relationship between EGFR gene copy number, EGFR protein expression, and clinical response to TKIs.

## DUAL PATHWAY INHIBITION

With clear evidence that targeted therapies can improve cancer patient outcomes, the concept of combining the activities of two targeted therapies in one treatment regimen for potentially synergistic activity is now under extensive investigation. Of particular interest is the simultaneous targeting of EGFR signaling plus other pathways involved in tumor development and progression, such as cell proliferation, apoptosis, and angiogenesis. Bevacizumab (Avastin®), a recombinant, humanized antibody, inhibits tumor angiogenesis by binding its ligand, VEGF. It is currently approved for treating mCRC and NSCLC (Avastin® [prescribing information], Genentech, South San Francisco, CA; 2005). In both NSCLC and SCCHN, angiogenesis has been strongly associated with tumor progression and poor outcomes. In a phase 2 study of patients with mCRC, cetuximab combined with bevacizumab ± irinotecan gave encouraging clinical activity and acceptable toxicity[53]. This same antibody combination is being investigated in two newer phase 2 trials: one involving patients with recurrent or metastatic SCCHN, and another in addition to paclitaxel and carboplatin, for patients with stage IIIb/IV NSCLC (www.clinicaltrials.gov).

The combination of erlotinib and bevacizumab appears feasible, with clinical activity in both NSCLC and SCCHN. A phase 1/2 trial enrolled 51 patients and administered erlotinib at a fixed dose of 150 mg in combination with escalating doses of bevacizumab to a maximum of 15 mg/kg. At the time of analysis, the ORR in the phase 2 cohort was 14.6%, with 19 patients achieving SD or better for six cycles or more. Median PFS was 3.8 months[54]. A phase 1 and 2 study of 40 patients with stage IIIb/IV NSCLC and at least one prior chemotherapy examined erlotinib plus bevacizumab[55]. Partial responses were observed in eight patients (20%), while 65.0% had SD as their best response. The 34 patients treated at the phase 2 doses showed a median OS of 12.6 months.

## DUAL KINASE OR MULTIKINASE ACTIVITY INHIBITORS

Novel agents with inhibitory activity against two different kinases have gained considerable interest, particularly with the approval of lapatinib, in combination with capecitabine, for patients with HER2-

positive, previously treated, metastatic breast cancer (Tykerb® [prescribing information], GlaxoSmithKline, Research Triangle Park, NC; 2007). A recent phase 2 study of lapatinib monotherapy in patients with recurrent or metastatic SCCHN showed a favorable side-effect profile, but little activity in EGFR–inhibitor-naive or -refractory patients[56]. This dual kinase inhibitor is currently being tested as adjuvant therapy in combination with radiotherapy and cisplatin to treat patients with locally advanced SCCHN in an international phase 3 trial.

Vandetanib is an orally available, small-molecule, dual TKI with specificity for vascular endothelial growth factor receptor (VEGFR)-2 and, at higher concentrations, for EGFR. Two phase 2 trials recently evaluated vandetanib's activity as a single agent and the feasibility of combining it with docetaxel in NSCLC[57,58]. Other studies are also under way to examine vandetanib in NSCLC in combination with pemetrexed, docetaxel, or paclitaxel and carboplatin.

## SUMMARY

Targeted therapies offer the promise of improved treatment with increased patient survival, lower toxicity, and higher QOL. EGFR-targeted therapies have resulted in disease stabilization and increased survival for patients with NSCLC and SCCHN, and they promise significant reduction in mortality and morbidity rates for these diseases. Cetuximab, erlotinib, and gefitinib, alone or in combination with chemotherapy, radiotherapy, or other targeted therapies, have all shown potential activity in both SCCHN and NSCLC; however, critical issues in study design and the evaluation of efficacy remain to be resolved. Pharmacogenomic screening for somatic mutations that can predict response of these tumors to gefitinib and erlotinib, and possibly other TKIs, may be useful in patient selection[39]. Other clinical characteristics (e.g., smoking history, performance status, and adenocarcinoma) are likely to be useful in determining treatment strategies and influencing outcomes. Predicting patient response based on severity of acne-like rash is also likely to prove useful in optimizing anti-EGFR treatment regimens for individual patients. To benefit fully from this new frontier of targeted therapy, future studies must address several parameters, including dosing regimens and schedules, and combinations with chemotherapeutics, radiation, and/or other targeted therapies.

## ACKNOWLEDGMENTS

Funding to RS is from NIH/NCI R01 (CA125541-02 and CA129501), American Lung Association, V-Foundation (with the Geleerd Family), Respiratory Health Association of Chicago, Kate McMullen Foundation, and the University of Chicago Cancer Research Center.
